# A Bipolar Clamp Mechanism for Activation of Jak-Family Protein Tyrosine Kinases

**DOI:** 10.1371/journal.pcbi.1000364

**Published:** 2009-04-17

**Authors:** Dipak Barua, James R. Faeder, Jason M. Haugh

**Affiliations:** 1 Department of Chemical and Biomolecular Engineering, North Carolina State University, Raleigh, North Carolina, United States of America; 2 Department of Computational Biology, University of Pittsburgh School of Medicine, Pittsburgh, Pennsylvania, United States of America; University of Georgia, United States of America

## Abstract

Most cell surface receptors for growth factors and cytokines dimerize in order to mediate signal transduction. For many such receptors, the Janus kinase (Jak) family of non-receptor protein tyrosine kinases are recruited in pairs and juxtaposed by dimerized receptor complexes in order to activate one another by trans-phosphorylation. An alternative mechanism for Jak trans-phosphorylation has been proposed in which the phosphorylated kinase interacts with the Src homology 2 (SH2) domain of SH2-B, a unique adaptor protein with the capacity to homo-dimerize. Building on a rule-based kinetic modeling approach that considers the concerted nature and combinatorial complexity of modular protein domain interactions, we examine these mechanisms in detail, focusing on the growth hormone (GH) receptor/Jak2/SH2-Bβ system. The modeling results suggest that, whereas Jak2-(SH2-Bβ)_2_-Jak2 heterotetramers are scarcely expected to affect Jak2 phosphorylation, SH2-Bβ and dimerized receptors synergistically promote Jak2 trans-activation in the context of intracellular signaling. Analysis of the results revealed a unique mechanism whereby SH2-B and receptor dimers constitute a bipolar ‘clamp’ that stabilizes the active configuration of two Jak2 molecules in the same macro-complex.

## Author Summary

Janus kinases (Jaks) interact with and activate receptors on the cell surface that mediate changes in gene expression. How these interactions are promoted and regulated is of central interest in fields such as cellular endocrinology and immunology. Here, detailed computational models of Jak activation are offered at the level of protein modification states and interaction domains, wherein the specification of only a handful of binding/reaction rules can produce networks comprised of thousands of differential equations. Specifically, we investigated the role of an adaptor protein, SH2-B, revealing a novel mechanism whereby it cooperates with receptors to form a stable complex that juxtaposes two Jak molecules for efficient activation. We refer to this mode of molecular assembly as the bipolar clamp mechanism.

## Introduction

Non-receptor protein tyrosine kinases of the Janus kinase (Jak) family play an essential role in signal transduction mediated by a host of cell surface receptors that lack intrinsic enzymatic activity. As a prominent example, the receptor for growth hormone (GH), a therapeutically important cytokine that modulates an array of cellular processes, including metabolism, proliferation, and survival [1], constitutively associates with intracellular Jak2 [2]–[4]. The ordered binding of the bivalent GH ligand results in the formation of active cell surface complexes comprised of one GH and two receptor molecules, a process that is understood in exquisite mechanistic detail [5]. The dimerized receptors juxtapose two associated Jak2 molecules, facilitating transphosphorylation of both Jak2 and the receptor [2]. Phosphorylation of Jak2 further activates the enzyme, and receptor phosphorylation sites foster recruitment of the signal transducer and activator of transcription (STAT) variants STAT3 and STAT5b, which are phosphorylated by Jak2 [6].

Given the central role of Jak2 in GH receptor signaling, it is not surprising that its function is modulated by other proteins. A prominent negative regulator is suppressor of cytokine signaling (SOCS)-1, which binds phosphorylated Tyr^1007^ in the activation loop of Jak2 and elicits degradation of the kinase [7],[8]. Conversely, the ubiquitously expressed adaptor protein SH2-Bβ also binds Jak2 but instead enhances its function [9]–[12]. The core structure of SH2-Bβ contains an N-terminal dimerization domain (DD), a pleckstrin homology (PH) domain, and a C-terminal Src homology-2 (SH2) domain. Among the multiple Jak2 sites phosphorylated in response to GH stimulation, Tyr^813^ is specifically recognized by the SH2-Bβ SH2 domain [13]. SH2-B also dimerizes by homotypic association of the DD, which has led to a conceptual model in which SH2-Bβ facilitates Jak2 autophosphorylation through formation of a heterotetrameric Jak2-(SH2-Bβ)_2_-Jak2 complex [14]. In support of this mechanism, purified SH2-Bβ enhances Jak2 phosphorylation in solution with a biphasic dose response, consistent with saturation of Jak2 at high SH2-Bβ concentrations to form dead-end Jak2-(SH2-Bβ)_2_ complexes; in the same study, it was further shown that either the SH2 domain or DD expressed alone can antagonize GH-stimulated Jak2 and STAT5b phosphorylation in cells [14]. There is also evidence to the contrary, as the SH2 domain of SH2-Bβ was sufficient to activate Jak2 in a different experimental context 15,16; if so, the biphasic dependence of Jak2 autophosphorylation on SH2-Bβ concentration might be attributed to a second, inhibitory interaction involving the PH domain. Although the PH domain has not yet been characterized fully, it has a speculated role in targeting SH2-Bβ to the plasma membrane, based on the established interactions of other PH domains with specific phosphoinositide lipids. Clearly, the two proposed mechanisms of SH2-Bβ function highlighted here present opposing views regarding the importance of DD dimerization.

**Figure 1:**
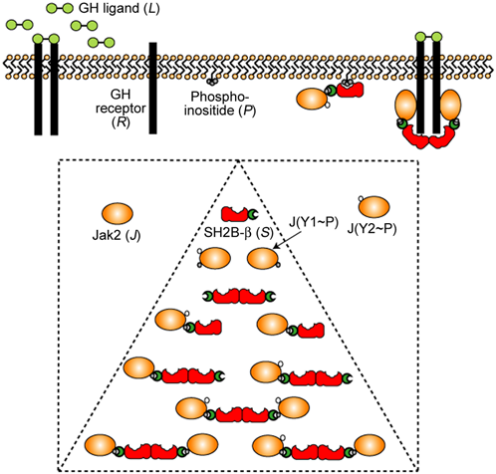
Molecular species and interactions considered in our models. Three models of increasing complexity were formulated and analyzed, as described under Methods. The In Vitro Model considers the enhancement of Jak2 (*J*) autophosphorylation by SH2-Bβ (*S*) in solution and includes 11 species (dashed triangle). Two Jak2 phosphorylation sites are considered: Y1, which when phosphorylated (Y1∼P) engages SH2-Bβ, and Y2, which when phosphorylated (Y2∼P) enhances the kinase activity. The model considers the best-case scenario where Y1 is constitutively (or rapidly) phosphorylated. The Simplified Cellular Model includes GH ligand (*L*) and GH receptor (*R*) and also considers Jak2 species with Y1 dephosphorylated in the cytosol (dashed square). In this model, Jak2 binds constitutively to receptors, but binding of two Jak2 molecules in the same complex is required for Jak2 autophosphorylation. Finally, the Extended Cellular Model additionally considers phosphoinositide (*P*) lipids, which mediate localization of SH2-Bβ to the plasma membrane. The complex shown in the upper right depicts the ‘bipolar clamp’ mechanism whereby SH2-Bβ stabilizes macro-complexes containing two Jak2 molecules.

In this work, we apply computational modeling to critically analyze the role of SH2-Bβ in Jak2 activation, revealing a novel mechanism. The model accounts for GH/GH receptor dynamics and Jak2/GH receptor, SH2/Jak2, DD/DD, and PH/lipid interactions in cells (Figure 1). As demonstrated in our previous domain-based models of Shp2 [17] and phosphoinositide 3-kinase regulatory subunit [18], this small number of interactions can produce thousands of distinct molecular species, and we manage this combinatorial complexity using the rule-based modeling approach [19]. Whereas our results challenge the notion that SH2-Bβ dimerization is sufficient for significant Jak2 association in solution or in cytosol, they also show that SH2-Bβ can significantly enhance Jak2 activation stimulated by GH. Dimerized receptors on the one hand, and dimerized SH2-B on the other, are proposed to act as a bipolar clamp that promotes Jak2 transphosphorylation by holding two Jak2 molecules in the same complex (Figure 1, top right).

## Results

### Jak2-SH2-Bβ heterotetramerization is an inefficient mechanism for promoting Jak2 autophosphorylation *in vitro*

**Figure 2:**
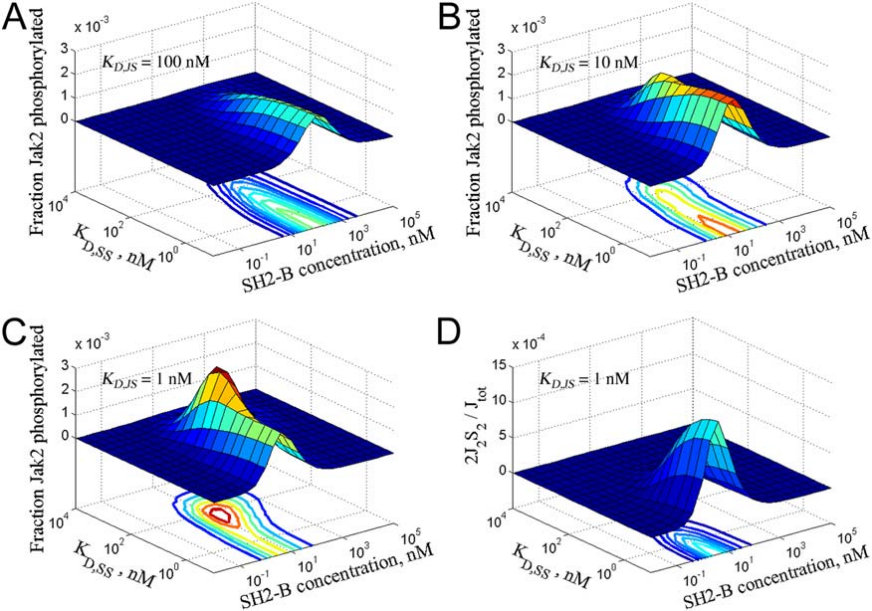
Critical analysis of the SH2-Bβ-mediated Jak2 autophosphorylation mechanism *in vitro*. (A–C) Surface and contour plots of Jak2 autophosphorylation (Y2∼P) for varying concentrations and dimerization *K_D_* values of SH2-Bβ, and with three different *K_D_* values of Jak2/SH2-Bβ binding: (A) *K_D,JS_* = 100 nM; (B) *K_D,JS_* = 10 nM; (C) *K_D,JS_* = 1 nM. See text for a description of the model assumptions, following [14]. (D) Surface and contour plot of heterotetramer (*JS*_2_ *J*) concentration for *K_D,JS_* = 1 nM.

Nishi et al. [14] purified Jak2 and SH2-Bβ and showed that SH2-Bβ enhances Jak2 autophosphorylation in solution. They obtained results with 14 pM Jak2 and SH2-Bβ concentrations in the range of 0.01–100 nM, which were incubated along with excess ATP for 10 minutes at 25°C in a total volume of 150 µL. The greatest change in Jak2 phosphorylation was seen as the SH2-Bβ concentration increased from 0.1 to 1 nM, and the effect of SH2-Bβ decreased at higher concentrations [14]. We recapitulated those conditions in our In Vitro Model (Methods), with the affinities of the SH2-Bβ (SH2)/Jak2 and SH2-Bβ dimerization (DD/DD) interactions varied systematically (Figure 2). The SH2 domain affinity, characterized by *K_D,JS_*, was assigned values in the range of 1–100 nM, which are at the low end of *K_D_* values (high affinity) measured for single SH2 domains [20],[21]. Indeed, although the *K_D_* of the interaction between full-length SH2-Bβ and Jak2 is not known, the isolated SH2 domain binds to a Jak2-derived phospho-peptide with *K_D_* = 80–550 nM [22],[23]. For DD dimerization, we considered an even wider range of *K_D,SS_* values, from 0.1 nM to 10 µM. Because there are no phosphatases present, the dephosphorylation reactions are turned off in the In Vitro Model, and as a best-case scenario, we assume that the SH2-Bβ binding site of Jak2 (Tyr^813^, or Y1) is pre-phosphorylated. In this context, phosphorylation of the Jak2 activation site (Tyr^1007^, or Y2) is the readout of the model, which serves as a surrogate for the modification of multiple Jak2 autophosphorylation sites.

The results show that, if Jak2 autophosphorylation were to proceed by the proposed heterotetramer (*JS*_2_ *J*) formation mechanism, the extent of phosphorylation is at most ∼0.3%, or <0.01 fmol, of Jak2 (Figure 2A–C). Analysis of the model indicates that the rate of phosphorylation is limited by the rate of exchange between phosphorylated and unphosphorylated Jak2 in the heterotetrameric complex, which is most affected by the rate of Jak2-SH2-Bβ association. The values of the association rate constants, *k_on,JS_* and *k_on,SS_*, are fixed at 0.06 nM^−1^ min^−1^ (1 µM^−1^ s^−1^) in the model, and therefore similar levels of Jak2 phosphorylation are predicted over multiple decades of *K_D_* (*k_off_*) values.

These results are difficult to reconcile with the experimental observations for the following reasons. First, to produce optimal phosphorylation at SH2-Bβ concentrations of ∼1–10 nM, extremely high-affinity interactions are required for both the SH2 domain and DD of SH2-Bβ (*K_D_* values ∼1 nM). Second, the predicted amount of phosphorylated Jak2 is probably too low to be detected by immunoblotting. Even if it were 10-fold higher, as by assuming *k_on_* = 10 µM^−1^ s^−1^ (quite high for protein-protein interactions), it is unclear whether or not it would be detectable.

The *in vitro* role of SH2-Bβ dimerization is even more difficult to reconcile if we relax the assumption that the SH2-Bβ binding site (Y1) is pre-phosphorylated. Indeed, an alternative model was considered that includes SH2-Bβ-independent Jak2 dimerization and phosphorylation of Y1 as a prerequisite for SH2-Bβ binding, and we found that very high concentrations of SH2-Bβ (≫100 nM) are needed to enhance Jak2 phosphorylation, even when the binding affinities are arbitrarily high; even then, the magnitude of the enhancement is quite small (Figure S1, Supporting Information). In that model, SH2-Bβ must associate rapidly with Jak2 dimers that happen to have catalyzed the phosphorylation of Y1 on both Jak2 molecules, but not of the activating site, Y2; Y2 phosphorylation on either Jak2 molecule leads to rapid phosphorylation of available sites, in which case SH2-Bβ binding has no bearing on the Jak2 phosphorylation status of that complex. With a total Jak2 concentration of 14 pM, the overall concentration of monomeric Jak2 with Y1 phosphorylated never achieves an appreciable concentration for dimerization of Jak2/SH2-Bβ complexes in solution.

Based on this analysis, the formation of *JS*_2_ *J* heterotetramers cannot adequately explain how SH2-Bβ apparently enhances Jak2 phosphorylation in this assay. The aforementioned alternative mechanism, whereby SH2-Bβ binding stabilizes Jak2 in a more active conformation [16], is more plausible in the context of Jak2 autophosphorylation in solution. In the rest of this paper, we focus on the more pertinent question of how SH2-Bβ dimerization might enhance Jak2 phosphorylation in cells.

### SH2-Bβ dimerization significantly enhances Jak2 autophosphorylation in the cellular context by coordinating Jak2/GH receptor binding: The bipolar clamp mechanism

**Figure 3:**
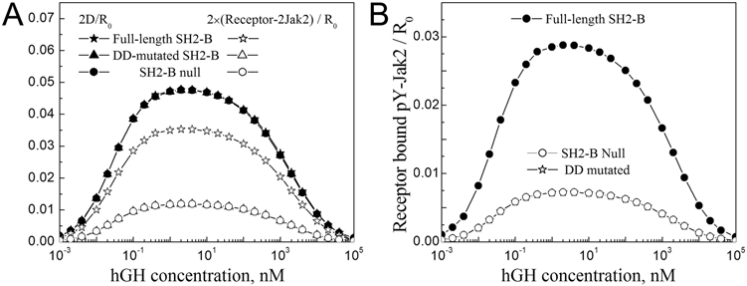
SH2-Bβ significantly enhances GH receptor-mediated Jak2 autophosphorylation *in vivo*. Steady-state calculations were performed using the Simplified Cellular Model, with equal Jak2 and SH2-Bβ concentrations and *K_D_* values (*J_Tot_* = *S_Tot_* = *K_D,JS_* = *K_D,SS_* = 100 nM). In the SH2-B null case, *S_Tot_* = 0, and in the DD-mutated SH2-B case, *K_D,SS_* = infinity; these two cases are functionally equivalent (and therefore the curves lie on top of one another). (A) SH2-Bβ does not affect GH dose-dependent receptor-dimerization (all of the filled symbols in panel A lie approximately on top of one another) but mediates ∼3-fold improvement in pair-wise recruitment of Jak2 to receptors (the number of Jak2 molecules engaged in receptor-Jak2 complexes containing two Jak2). (B) Accordingly, SH2-Bβ enhances Jak2 autophosphorylation (site Y2) by roughly 3-fold.

Whereas it seems unlikely that SH2-Bβ-mediated heterotetramers could form to a significant extent in solution to explain the activation of Jak2 *in vitro*, Jak2 kinase activity is normally associated with cytokine receptor signaling at the plasma membrane *in vivo*. Using our Simplified Cellular Model (Methods), we quantified activated (receptor-bound and Y2-phosphorylated) Jak2 stimulated by varying doses of GH at steady state, relative to the number of cell-surface GH receptors in the absence of GH (Figure 3); as explained previously [24], maximal GH receptor activation is accompanied by significant downregulation from the surface, so a relative value of ∼0.05 by this measure is the maximum. The Simplified Cellular Model does not allow for membrane localization of SH2-Bβ through its PH domain.

In the absence of SH2-Bβ, or (equivalently) with SH2-Bβ lacking the DD, the Jak2/receptor binding may be estimated in a straightforward manner. For the parameter values assumed, with total Jak2 expression in excess over receptors and equal to the *K_D_* of Jak2/receptor binding, roughly half of the dimerized receptors are bound with Jak2, and so roughly 1/4 of the receptor dimers have two Jak2 molecules bound and phosphorylated at steady state (Figure 3A). It is noted that, for the parameter values assumed, the two Jak2 molecules remain almost fully phosphorylated on Y1 and Y2 while in the same receptor complex; therefore, allowing SH2-Bβ binding to further enhance Jak2 catalytic activity [16] is of little consequence in this context (Figure S2A, Supporting Information). By comparison, the presence of dimerization-competent SH2-Bβ (with the reasonable assumption that *S_Tot_* = *J_Tot_* = *K_D,JS_* = *K_D,SS_*) increases by ∼3-fold the number of receptor dimers with two Jak2 bound (Figure 3A) and, accordingly, the number of Jak2 molecules with Y2 phosphorylated (Figure 3B). Analysis of the model shows that it does so by forming stable, seven-member “macro-complexes” containing GH, two receptor, two Jak2, and two dimerized SH2-Bβ molecules, as depicted in Figure 1. Thus, dimerized receptors serve as a template for Jak2 recruitment, and, once Jak2 has been autophosphorylated, the SH2-Bβ dimer clamps the active Jak2 molecules in place.

**Figure 4:**
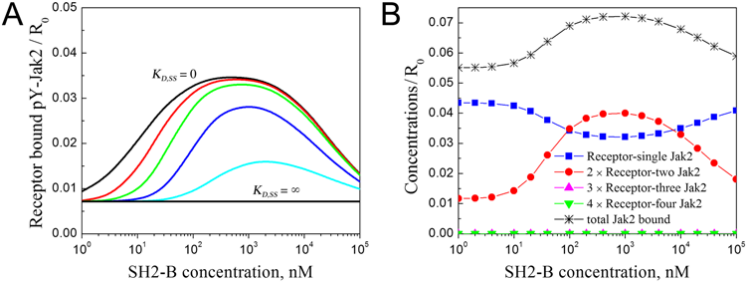
SH2-Bβ dimerization coordinates the formation of macro-complexes containing two Jak2 molecules bound to GH-dimerized receptors. Steady-state calculations were performed using the Simplified Cellular Model and the same parameter values as in Figure 3, except with 10 nM GH stimulation and varying SH2-Bβ concentration. (A) Receptor-bound, phosphorylated Jak2 (Y2∼P), for various values of the SH2-Bβ dimerization affinity. The extreme cases of *K_D,SS_* equal to zero and infinity correspond to irreversible and no dimerization, respectively; intermediate *K_D,SS_* values are 10 nM, 100 nM, 1 µM, and 10 µM. (B) Analysis of receptor/Jak2 complexes, with *K_D,SS_* = 100 nM. SH2-Bβ dimerization coordinates the binding of two Jak2 molecules to dimerized receptors, while affecting overall receptor/Jak2 binding only modestly. Complexes containing more than two Jak2 molecules (e.g., *J*(*RLR*)*JS*_2_ *J*) are rare.

To further characterize this hypothetical mechanism, the intracellular concentration and dimerization affinity of SH2-Bβ were varied for a constant GH concentration of 10 nM (Figure 4). Although a broad range of SH2-Bβ concentrations was tested in order to evaluate the full spectrum of behaviors, it is noted that the endogenous SH2-Bβ expression level is not expected to be above the nanomolar range. Given a constant Jak2/SH2-Bβ affinity (*K_D,JS_* = 100 nM), the SH2-Bβ concentration should be of a similar magnitude or somewhat higher for near maximal enhancement of Jak2 phosphorylation; extremely high SH2-Bβ concentrations, similar in magnitude to *χ_r_* (100 µM; see Methods) are needed to antagonize the formation of the stable macro-complex, leading instead to formation of less stable, nine-member *S*_2_ *J*(*RLR*)*JS*_2_ complexes (Figure 4A). Analysis of the GH receptor/Jak2 complexes formed reveals that, as expected, SH2-Bβ stabilizes complexes with two Jak2 molecules while increasing the total Jak2 recruitment only modestly (Figure 4B). In the absence of SH2-Bβ, approximately half of all GH receptors are Jak2-bound, and this constitutive binding accounts for a significant fraction of the total at all SH2-Bβ concentrations.

### Membrane localization of SH2-Bβ via its PH domain broadens Jak2 activation potency, but SH2-Bβ dimerization is still essential

**Figure 5:**
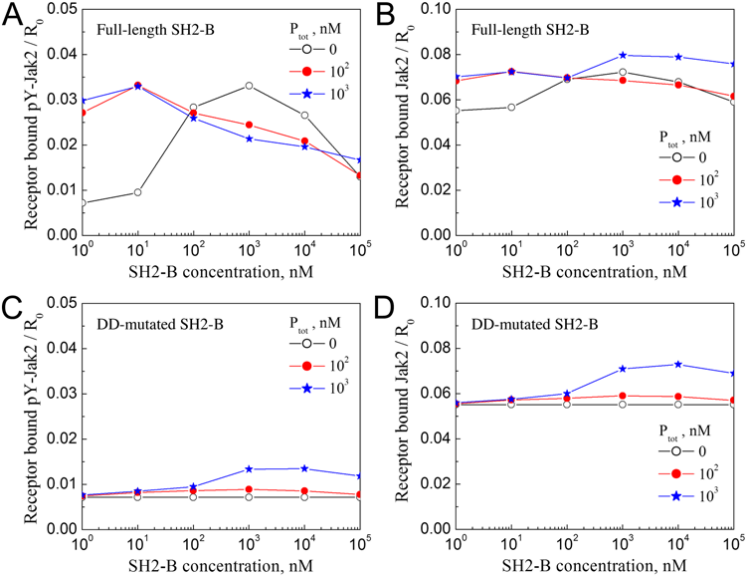
Membrane localization and dimerization of SH2-Bβ synergize to enhance the potency of its Jak2 activation-promoting function. Steady-state calculations were performed using the Extended Cellular Model and the same parameter values as in Figure 4B. The total concentration of phosphoinositide, on a whole-cell basis, is either 0, 100 nM, or 1 µM as indicated, and its recruitment of SH2-Bβ PH domain is characterized by *K_D,SP_* = 100 nM. Two scenarios are considered: full-length SH2-Bβ (A and B) and SH2-Bβ with the dimerization domain absent (C and D). The calculated quantities are receptor-bound, phosphorylated Jak2 (Y2∼P; A and C) and total receptor-bound Jak2 (B and D).

We next considered the role of the SH2-Bβ PH domain, which is thought to mediate binding with phosphoinositides and thus plasma membrane localization [25], in our Extended Cellular Model (Figure 5). Based on physical principles, membrane localization increases the rate of association between complexes containing receptor or/and phosphoinositide molecules by roughly two orders of magnitude, enhancing the binding of SH2-Bβ with receptor-bound Jak2 (Methods). In fact, we find that the addition of the PH domain interaction broadens the efficacy of SH2-Bβ-mediated Jak2 activation down to low nanomolar SH2-Bβ concentrations, well below the assumed *K_D_* of the Jak2/SH2-Bβ interaction in solution (Figure 5A). As in the Simplified Cellular Model, this enhancement is not accompanied by dramatic gains in overall Jak2/receptor binding (Figure 5B). Membrane localization of SH2-Bβ facilitates binding to receptor-bound Jak2 and SH2-Bβ dimerization, and therefore it stabilizes signaling-competent macro-complexes at the expense of other receptor/Jak2 complexes.

To probe this mechanism further, we repeated the analysis with the DD of SH2-Bβ removed. Intuitively, one might expect that membrane localization of SH2-Bβ would drive significantly more Jak2 into complex with receptors; however, this was not the case with the DD present (Figure 5B), and accordingly, Jak2 autophosphorylation was not dramatically enhanced by SH2-Bβ with the DD absent, even with arbitrarily high SH2-Bβ and phosphoinositide concentrations (Figure 5C and 5D). Variation of the other parameters, such as the Jak2 concentration and binding affinities, did not qualitatively affect the outcome (results not shown).

Why is SH2-Bβ dimerization predicted to be so important in the cellular context? A key insight is that Jak2 must be phosphorylated on Y1, by associating with dimerized receptors, before it can bind membrane-localized SH2-Bβ. Phosphorylated Jak2 might even associate with SH2-Bβ quite readily, but the lifetime of the receptor/Jak2 interaction is not affected as a result. The association of *JSP* complexes with free receptors is modest because this pool of Jak2 is small; once formed, the *JSP* complex is more likely to dissociate via one of its two linkages than to associate with a free receptor site, and when it does bind free receptors, it does not discriminate between dimerized and inactive receptor molecules. By comparison, SH2-Bβ dimerization specifically stabilizes Jak2 interactions with dimerized receptors; this is the essence of the bipolar clamp mechanism.

### Predictions regarding the potency of SH2-Bβ mutants as dominant-negative inhibitors of GH receptor signaling

**Figure 6:**
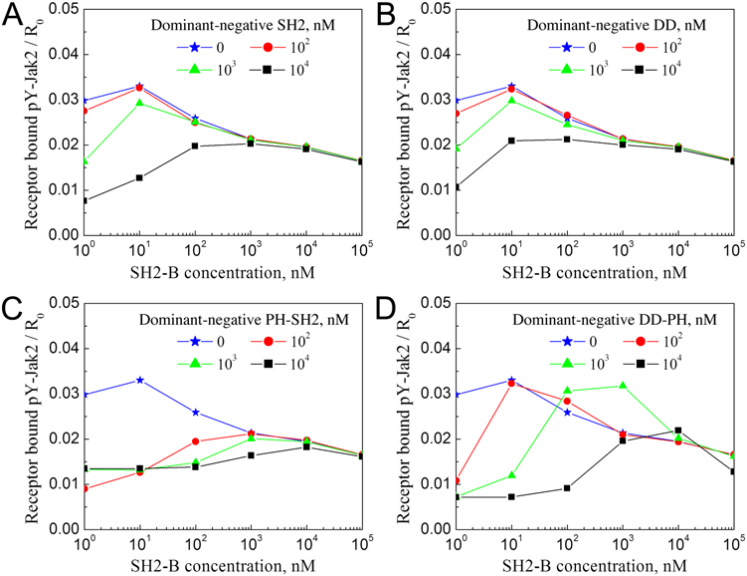
Potencies of SH2B-β domain mutants as dominant negatives antagonizing wild-type SH2-Bβ function. Jak2 phosphorylation was calculated using the Extended Cellular Model as in Figure 5A, with the same parameter values and *P_Tot_* = 1 µM. To this model, we added one of the following SH2-Bβ constructs: SH2 only (A), DD only (B), PH-SH2 (C), and DD-PH (D). As indicated, the value of the overall inhibitor concentration was either 0 (no inhibition; same as Figure 5A), 100 nM, 1 µM, or 10 µM.

To further evaluate the roles of the functional SH2-Bβ domains, we assessed the ability of different domain mutants to antagonize the function of wild-type SH2-Bβ in cells, i.e., to act as a dominant negative (Figure 6). The Extended Cellular Model was used with the addition of the mutant SH2-Bβ species. The SH2 domain alone competes with wild-type for Jak2 binding and is an effective inhibitor at concentrations of at least 1 µM (for nanomolar concentrations of endogenous SH2-Bβ, as expected), which is 10-fold higher than the assumed value of *K_D,JS_* (Figure 6A). Inhibition by the DD alone is through dimerization with wild-type SH2-Bβ and is somewhat less effective (Figure 6B), which might be attributed to the partial neutralization of the DD through homo-dimerization. The addition of the PH domain to either the SH2 domain (functionally equivalent to the DD-mutated SH2-B analyzed in Figure 5C and 5D) or the DD results in membrane localization of the mutant SH2-Bβ and, accordingly, more potent disruption of receptor/Jak2/SH2-Bβ macro-complexes when it is expressed in excess compared with wild-type SH2-Bβ; comparing PH-SH2 and DD-PH, the former construct shows the more robust inhibition of SH2-Bβ function (Figure 6C and 6D). The predicted efficacies of these two dominant-negatives reflect the gamut of effects, both strong and subtle, discussed previously: 1) the effect of SH2-Bβ concentration, relative to its Jak2-binding affinity, on macro-complex formation; 2) antagonism of macro-complex formation at extreme SH2-Bβ concentrations, exceeding the value of *χ_r_*; and 3) the ability of phosphoinositides to enhance the effective concentration of SH2-Bβ, which facilitates macro-complex formation at low SH2-Bβ concentrations and also a modest degree of Jak2-receptor association at high SH2-Bβ concentrations that is independent of SH2-Bβ dimerization.

## Discussion

This is the third system we have studied using the rule-based modeling approach to specifically address the concerted binding of multiple, modular domains in signaling proteins. This aspect of signal transduction is a recognized source of complexity in the signal transduction field [26],[27], yet it is commonly side-stepped in the formulation of mathematical models of signaling pathways. At the level of pathways and networks, we recognize and espouse that the finer molecular details, while important to consider, must be simplified (or “lumped”, in the mathematical sense). The rule-based approach addresses the problem of combinatorial complexity [28], its main strength being that it allows the modeler to invoke more mechanistic or biologically plausible assumptions [29]; however, it cannot ease the burden associated with specifying a large number of model parameters, which becomes increasingly problematic at the pathway/network level. For this reason, we apply rule-based modeling to subsystems that involve only a handful of interactions yet give rise to combinations of complexes that could not readily be enumerated in the classical way. Indeed, in this work, models with as many as 3,821 differential equations were generated. Despite their large size and complex structure, these models were generated with a small number of generating equations (“rules”) and are governed by only a handful of parameters.

We analyzed the receptor-mediated activation of Jak2 and the role of the adaptor protein SH2-Bβ, which contains three modular domains (DD, PH, and SH2), and demonstrated how modeling can be used to evaluate the integration of domain functions as they affect receptor-mediated signaling in cells. In particular, we sought to clarify the role of SH2-Bβ dimerization. Protein homodimerization, or dimerization of structurally homologous proteins, is a ubiquitous process in molecular biology and permeates signal transduction from the receptor level (e.g., cytokine receptors, receptor tyrosine kinases) to the activation of transcription factors (e.g., STATs, Smads). Ligand-induced dimerization of the GH receptor is necessary but not sufficient for intracellular signaling, requiring also the juxtaposition of two Jak2 molecules; this theme is common to (and our conclusions are predicted to be applicable to) signaling mediated by dimers of the closely-related erythropoietin receptor [30]. Dimerization of SH2-B isoforms, and of the closely related APS proteins, is unique because they are considered adaptors or modulators of, not executors of, intracellular signaling.

Our results suggest that dimerization of SH2-B goes hand in hand with the binding of Jak2 to dimerized receptors, which template the assembly of the *JS*_2_ *J* heterotetrameric unit. Thus, dimerized receptors and SH2-Bβ together coordinate the recruitment of two Jak2 molecules. At least in the context of our models, it is incorrect to characterize SH2-Bβ dimerization as a means of bringing two Jak2 molecules together, as might be inferred by the ability of the adaptor to enhance Jak2 autophosphorylation in solution; rather, we suggest that it acts as a clamp that stabilizes existing *J*(*RLR*)*J* complexes. This is because Jak2 must already be autophosphorylated, at least on Tyr^813^, for SH2-Bβ to bind. Accordingly, enhancing the association rates of the RJ/S or R/JS linkages, as by membrane localization of SH2-Bβ, is insufficient for significant enhancement of Jak2 phosphorylation if SH2-Bβ cannot dimerize.

This work puts forward a number of testable predictions. One concerns the mechanism by which SH2-Bβ dimerization affects Jak2 autophosphorylation, as outlined above. We anticipate that testing the bipolar clamp mechanism concept would be a challenge, because for any mechanism involving SH2-Bβ, enhancement of GH-stimulated Jak2 phosphorylation hinges upon Jak2 binding to GH receptors and its subsequent phosphorylation on Tyr^813^. In principle, however, one could express the following Jak2 mutants in cells, in parallel experiments: 1) a phosphorylation-mimicked (Y813D) Jak2 mutant, 2) a phosphorylation-deficient (Y813F) Jak2 mutant, and 3) a variant of mutant 1 that cannot interact with GH receptor. If over-expressed at a level that is sufficient to saturate constitutive binding to GH receptors, where applicable, then cells expressing mutant 1 or mutant 2 (or wild-type Jak2) would be expected to show similar levels of GH-stimulated autophosphorylation, greater than those expressing mutant 3, because the stabilizing effect of SH2-Bβ would be superfluous. In contrast, if mutant 1 and mutant 2 were co-expressed in cells at similar levels, SH2-Bβ should stabilize mutant 1 (or wild-type Jak2) relative to mutant 2, which would be reflected by their differential autophosphorylation.

Other predictions consider the potential role of the SH2-Bβ PH domain (or whichever structural motif is responsible for the observed membrane localization). In a cellular context where endogenous SH2-Bβ expression is lacking or repressed, comparison of wild-type SH2-Bβ and a mutant defective in lipid binding might only show moderate differences, and in fact the mutant might outperform the wild-type adaptor if the adaptor concentration is in the high nanomolar range (as is often the case for expression plasmids; Figure 5). The model results suggest that the role of membrane localization is to broaden the efficacy of SH2-Bβ to low or sub-nanomolar concentrations of the adaptor. But by the same token, we show that membrane localization of SH2-Bβ should enhance the inhibitory properties of constructs that lack either the SH2 domain or the DD, and thus the importance of the membrane localization effect might be more effectively interrogated through such inhibition experiments. To put these predictions in the proper context, it will be important to identify the sequence(s) of SH2B-β responsible for its apparent membrane localization, whether in the PH domain or elsewhere in the molecule.

Besides the bipolar clamp mechanism explored here, it has also been postulated that SH2-Bβ binding is sufficient for enhancing Jak2 catalytic efficiency [16], and this alternative mechanism might account for the apparent ability of SH2-Bβ to enhance Jak2 autophosphorylation in solution and in unstimulated cells (with Jak2 overexpressed) [14]. This alternative mechanism might also complement the bipolar clamp function of SH2-Bβ, but only in cells where Jak2 in *J*(*RLR*)*J* complexes (without SH2-Bβ bound) tends to be dephosphorylated, at least on certain sites (it is important to bear in mind that Tyr^813^ must be autophosphorylated in order for SH2-Bβ to bind; see Figure S2B, Supporting Information). Likewise, there are intracellular conditions in which the clamping function would have no apparent effect; as implied above, saturation of constitutive Jak2-receptor binding renders the mechanism unnecessary. As we have found for other proteins with multiple binding domains, there is a clear indication that the function of SH2-Bβ, and even the dominant mechanism by which it functions, is context-dependent.

Finally, we speculate that the bipolar clamp mechanism studied here for the Jak2/SH2-B system will be applicable to analogous signal transduction processes. A striking example is that of 14-3-3 proteins [31]. Like proteins of the SH2-B/APS family, 14-3-3 proteins lack catalytic function, they homo- and hetero-dimerize, and they simultaneously engage certain phosphorylated proteins (on phospho-serine/-threonine rather than phospho-tyrosine). Indeed, 14-3-3 proteins are thought to promote the formation of complexes containing two isoforms of Raf [32], serine/threonine kinases that function in the most prominently studied of the mitogen-activated protein kinase cascades. This provides a clue that functionally similar mechanisms might be at play at multiple points of signal transmission from the cell surface to the nucleus.

## Methods

### Base model of GH/GH receptor dynamics

Where applicable, we build upon a previous model of GH/GH receptor interactions and trafficking [24] and use the same parameter values for wild-type human GH. Briefly, the GH ligand concentration [*L*] is fixed and is an input variable to the model, and unbound GH receptors (*R*) are present at a level of 2×10^3^ molecules/cell initially. Receptor expression is determined by the ratio of the synthesis rate [*V_s_* = 10 (#/cell)/min] and basal turnover rate constant (*k_t_* = 0.005 min^−1^). Ligand-receptor complexes (*C*) form with site 1 forward rate constant *k_f_*_1_ = 0.1 nM^−1^ min^−1^ and reverse rate constant *k_r_*_1_ = 0.15 min^−1^ and are subject to basal turnover. Receptor dimers (*D*), which are competent for signaling, form from *C* and *R* with site 2 forward rate constant *k_x_*_2_ = 2.42×10^−3^ (#/cell)^−1^min^−1^ and reverse rate constant *k*_−*x*2_ = 0.016 min^−1^, and they can also dissociate via the site 1 linkage with rate constant 1.5×10^−3^ min^−1^ (as noted previously, setting this rate equal to zero does not affect the results for wild-type human GH), leaving the ligand to dissociate rapidly via the unstable site 2 linkage. Dimers are endocytosed and degraded at an enhanced rate, with rate constant *k_e_* = 0.1 min^−1^. Secondary effects of Jak2 and SH2-Bβ interactions on GH/GH receptor dynamics are discussed below.

### Intracellular interactions: General considerations

Our models are based on mass-action kinetics, with bimolecular (association of two species) and unimolecular (dissociation or change in state of a complex) transitions. Definitions and ranges of values for the model parameters are given in Table S1 (Supporting Information). For all bimolecular interactions where one or both of the species is in the cytosol, the association rate constant *k_on_* was assigned a typical value of 0.06 nM^−1^ min^−1^ (or 1.0 µM^−1^ s^−1^) [33], and the dissociation rate constant *k_off_* is calculated from *k_off_* = *k_on_ K_D_*, where *K_D_* is the specified equilibrium dissociation constant. The total intracellular concentrations of Jak2, SH2-Bβ, and phosphoinositide (*J_Tot_*, *S_Tot_*, and *P_Tot_*, respectively) are conserved and are specified alternatively in units of molar concentration or molecules/cell; these units are interconverted by assuming a volume of 0.52 pL, equivalent to that of a sphere with 5 µm radius.

### Jak2 phosphorylation

Jak2 binds receptors, regardless of their ligand-bound status and the phosphorylation status of Jak2, with a *K_D_* defined as *K_D,RJ_*. The model considers phosphorylation of two Jak2 tyrosine sites, Y1 and Y2, corresponding to Tyr^813^ and Tyr^1007^, which are responsible for SH2-Bβ association and stimulated activation of Jak2 kinase activity, respectively. Consistent with the current understanding of GH receptor activation, Jak2 can be phosphorylated on Y1 and Y2 only when two Jak2 molecules are associated with the same complex (receptor or/and SH2-Bβ mediated). Once Y2 is phosphorylated, the catalytic efficiency of that Jak2 molecule increases substantially. Accordingly, we model Jak2 phosphorylation as a pseudo-first order process, and once Y2 of the Jak2 molecule acting as the enzyme is phosphorylated, its phosphorylation rate constant towards both Y1 and Y2 of the other Jak2 molecule increases from 6 min^−1^ (0.1 s^−1^) to 60 min^−1^ (1 s^−1^). Jak2 dephosphorylation is also modeled as a pseudo-first order process, with a rate constant of 6 min^−1^ for both Y1 and Y2; phosphorylated Y1 that is bound to SH2-Bβ is protected from dephosphorylation.

### Interactions involving SH2-Bβ

SH2-Bβ participates in as many as three interactions, with *K_D_* values defined as follows: its SH2 domain binds to Jak2 molecules with Y1 phosphorylated (*K_D,JS_*), its DD dimerizes (*K_D,SS_*), and its PH domain binds phosphoinositides (*K_D,SP_*).

The introduction of SH2-Bβ in the system gives rise to interactions in the plane of the membrane or within a multi-molecular complex, and these occur at accelerated rates in the forward direction as compared to the situation where one or both of the interacting species is in the cytosol. Dissociation of such a linkage is assumed to occur with the same rate constant as when one or both of the dissociating components is/are released into the cytosol.

Interactions between two membrane-associated species arise as a consequence of SH2-Bβ binding to phosphoinositide lipids (*PS*) or to receptor-bound Jak2 (*RJS*, with or without ligand), which can subsequently form complexes such as *PS*_2_ *P*, *RJSP*, *RJS*_2_ *JR*, etc. To simplify the model in a manner that satisfies detailed balance, interactions in the membrane are assigned a forward rate constant that is calculated as *χ_m_k_on_*, with *k_on_* = 0.06 nM^−1^ min^−1^ = 1.91×10^−4^ (#/cell)^−1^min^−1^ and *χ_m_* defined as a common, dimensionless enhancement factor; as considered in previous signal transduction models [34],[35], its value is based on a confinement layer (reduced volume) with 10 nm thickness at the membrane, yielding *χ_m_* = [(5 µm)/3(10 nm)](10^3^ nm/µm) = 167. The corresponding dissociation rate constant is assumed to be the same as for release of one or both species to the cytoplasm; this assumption could be relaxed if diffusion limitations were to be considered.

Interactions within a complex (ring closure) include the association of two SH2-Bβ molecules with dangling DDs, as in the species *SJ*(*RLR*)*JS*, or association of SH2-Bβ and Jak2 in the *J*(*RLR*)*JS*_2_ complex, for example. Ring closure is a unimolecular transition with forward rate constant calculated as *χ_r_k_on_*, where *χ_r_* is the effective concentration of an unbound site within the complex, assumed to be the same for all such interactions (the notation is from [35], referring to interactions within a receptor complex). A conservative value of *χ_r_* = 100 µM was used (see [18] for a detailed discussion). Ring closure also affects GH binding, because of the ability of the *JS*_2_ *J* heterotetramer to dimerize receptors without ligand present. Thus, the model accounts for closure of species such as *LRJS*_2_ *JR* via the GH (site 2)/GH receptor linkage; because GH-induced receptor dimerization normally occurs in the plane of the membrane, the association rate constant for this ring closure transition is calculated as (*χ_r_*/*χ_m_*)*k_x_*_2_.

To avoid the formation of potentially infinite chains at the membrane, which would occur if GH/GH receptor dimers were clustered via *JS*_2_ *J* linkages (which would be a rare occurrence if accounted for), the model is constrained so that complexes may contain no more than 2 receptor molecules. All complexes containing 2 receptors, whether they contain ligand or not, are considered receptor dimers and are subject to enhanced endocytosis, with rate constant *k_e_* = 0.1 min^−1^. Internalized receptors cannot associate with Jak2; any Jak2 and SH2-Bβ in complex with a receptor when it is internalized (whether endocytosed by the induced or basal turnover pathway) dissociate at the normal rate.

### Specific model cases and rule-based model implementation

Our simplest model is the so-called In Vitro Model, which contains only Jak2 and SH2-Bβ molecules, and therefore the largest complex in this model is the heterotetramer, *JS*_2_ *J*. It considers the best-case scenario where all Y1 sites are pre-phosphorylated and thus generates only 11 species (state variables) (Figure 1). The dephosphorylation reactions are turned off in the In Vitro Model, because phosphatases are not present. The Simplified Cellular Model considers all of the interactions except those with phosphoinositides, generating 470 species (5,033 reactions). The Extended Cellular Model adds the influence of phosphoinositides and generates 2,561 distinct species (41,233 reactions). In variations of this model, we also considered the influence of a SH2-Bβ mutant lacking one or two of its domains, acting as a dominant negative, alongside the wild-type SH2-Bβ species; these yielded even more species and reactions, according to the complexity of the dominant negative construct considered: SH2, 2,849 species; DD, 3,154 species; PH-SH2, 3,152 species; DD-PH, 3,821 species.

Our rule-based model was developed using the software program BioNetGen2, which is freely available through http://bionetgen.org. As discussed in detail elsewhere [17],[36], the user defines the biochemical network in terms of molecules, their interaction domains, and context-dependent rules for association/dissociation or covalent modification. Based on those rules, an exhaustive search is performed to automatically generate all possible species (combinations of interactions and modification states) and their corresponding conservation equations (differential equations in time), which are numerically integrated using a standard stiff solver up to time = 10^3^ min, by which time the system was confirmed to have reached steady state. For the In Vitro Model, a time of 10 min was used, corresponding to the experimental conditions. The BNGL files specifying the rules for the In Vitro, Simplified Cellular, and Extended Cellular models have been included in the online Supporting Information (Text S1, Text S2, and Text S3, respectively); note that some of the parameter values were varied as indicated in the figure legends.

## Supporting Information

Figure S1 Jak2 autophosphorylation in vitro without pre-phosphorylation of the SH2-Bβ binding site. Surface and contour plots of Jak2 autophosphorylation (Y2∼P) for varying concentrations and dimerization *K_D_* values of SH2-Bβ, and with three different *K_D_* values of Jak2/SH2-Bβ binding, following the experimental conditions reported by Nishi et al. Here, unlike the results presented in Figure 2, the SH2-Bβ binding site (Y1) is not pre-phosphorylated. Rather, Jak2 is allowed to dimerize in the absence of SH2-Bβ (with *k_on_* = 1 µM^−1^ s^−1^ and *K_D_* = *k_off_*/*k_on_* = 100 nM), which must happen if Y1 is to be phosphorylated. Under these conditions, SH2-Bβ has very little effect (note the scale of the *z*-axis); if Y1 has been phosphorylated, it is likely that Y2 has been phosphorylated as well, in which case SH2-Bβ binding has no bearing on the Jak2 phosphorylation status of that complex.
(0.20 MB PDF)

Figure S2 Considering the enhancement of Jak2 catalytic activity by SH2-Bβ binding. Calculations were performed using the Simplified Cellular Model as outlined under Figure 3, except an additional effect was added: Jak2 with SH2-Bβ bound was assumed to have 10-fold higher catalytic activity towards other Jak2 molecules in the same complex. Note here that the absence of SH2-Bβ (SH2-B null) and the inability of SH2-Bβ to dimerize (DD-mutated) are not equivalent. (A) Jak2 with SH2-Bβ bound has catalytic rate constants that are 10 times greater than the base values (i.e., *k_phos,slow_* = 60 min^−1^, *k_phos,fast_* = 600 min^−1^). Under those conditions, there is no significant effect on Jak2 phosphorylation (compare to Figure 3), because the catalytic activity is already high enough so that two Jak2 molecules remain almost fully phosphorylated on Y1 and Y2 while in the same receptor complex. (B) Jak2 without SH2-Bβ bound has catalytic rate constants that are 10 times lower than the base values (i.e., *k_phos,slow_* = 0.6 min^−1^, *k_phos,fast_* = 6 min^−1^). In this case, Jak2 is not sufficiently phosphorylated on Y1, the SH2-Bβ binding site, and therefore does not enjoy the benefit of SH2-Bβ enhancement of Jak2 catalytic activity.
(0.23 MB PDF)

Table S1 Model parameters.
(0.09 MB PDF)

Text S1 BNGL code for the In Vitro Model
(<1 MB TXT)

Text S2 BNGL code for the Simplified Cellular Model
(0.01 MB TXT)

Text S3 BNGL code for the Extended Cellular Model
(0.01 MB TXT)
