# Supplementary material for: A Bipolar Clamp Mechanism for Activation of Jak-Family Protein Tyrosine Kinases
Source: PLoS Comput Biol. 2009 Apr 17;5(4):e1000364. doi: 10.1371/journal.pcbi.1000364 (PMC2667146; doi:10.1371/journal.pcbi.1000364)
Supplement: Figure S1 — Jak2 autophosphorylation in vitro without pre-phosphorylation of the SH2-Bβ binding site. Surface and contour plots of Jak2 autophosphorylation (Y2∼P) for varying concentrations and dimerization KD values of SH2-Bβ, and with three different KD values of Jak2/SH2-Bβ binding, following the experimental conditions reported by Nishi et al. Here, unlike the results presented in Figure 2, the SH2-Bβ binding site (Y1) is not pre-phosphorylated. Rather, Jak2 is allowed to dimerize in the absence of SH2-Bβ (with kon = 1 µM−1 s−1 and KD = koff/kon = 100 nM), which must happen if Y1 is to be phosphorylated. Under these conditions, SH2-Bβ has very little effect (note the scale of the z-axis); if Y1 has been phosphorylated, it is likely that Y2 has been phosphorylated as well, in which case SH2-Bβ binding has no bearing on the Jak2 phosphorylation status of that complex. (0.20 MB PDF) [file pcbi.1000364.s001.pdf]

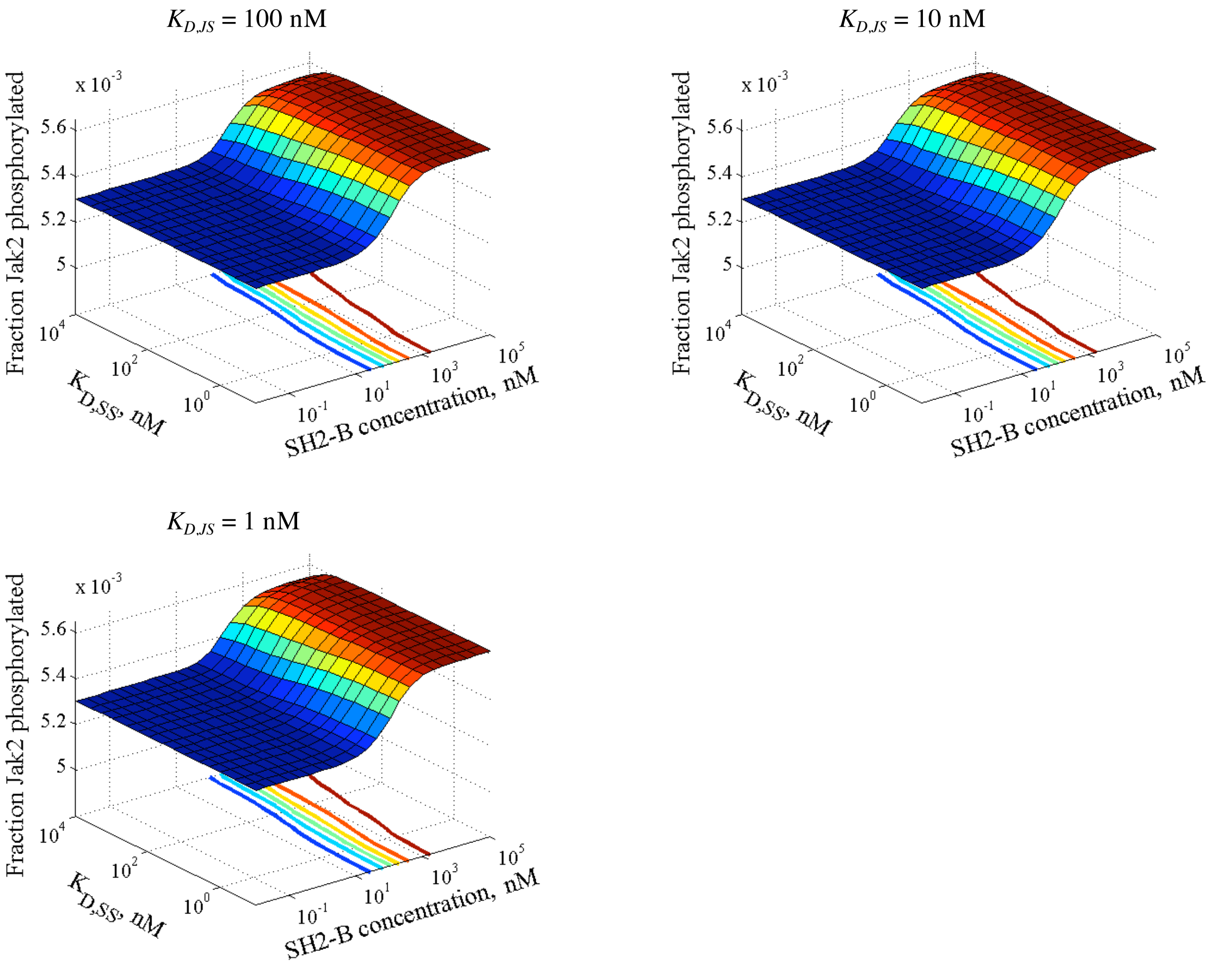

FIGURE S1. Jak2 autophosphorylation *in vitro* without pre-phosphorylation of the SH2-Bβ binding site. Surface and contour plots of Jak2 autophosphorylation (Y2~P) for varying concentrations and dimerization K_D values of SH2-Bβ, and with three different K_D values of Jak2/SH2-Bβ binding, following the experimental conditions reported by Nishi et al. Here, unlike the results presented in Fig. 2, the SH2-Bβ binding site (Y1) is not pre-phosphorylated. Rather, Jak2 is allowed to dimerize in the absence of SH2-Bβ (with k_{on} = 1 $\mu M^{-1}s^{-1}$ and $K_D = k_{off}/k_{on}$ = 100 nM), which must happen if Y1 is to be phosphorylated. Under these conditions, SH2-Bβ has very little effect (note the scale of the *z*-axis); if Y1 has been phosphorylated, it is likely that Y2 has been phosphorylated as well, in which case SH2-Bβ binding has no bearing on the Jak2 phosphorylation status of that complex.
