# Supplementary material for: A Bipolar Clamp Mechanism for Activation of Jak-Family Protein Tyrosine Kinases
Source: PLoS Comput Biol. 2009 Apr 17;5(4):e1000364. doi: 10.1371/journal.pcbi.1000364 (PMC2667146; doi:10.1371/journal.pcbi.1000364)
Supplement: Figure S2 — Considering the enhancement of Jak2 catalytic activity by SH2-Bβ binding. Calculations were performed using the Simplified Cellular Model as outlined under Figure 3, except an additional effect was added: Jak2 with SH2-Bβ bound was assumed to have 10-fold higher catalytic activity towards other Jak2 molecules in the same complex. Note here that the absence of SH2-Bβ (SH2-B null) and the inability of SH2-Bβ to dimerize (DD-mutated) are not equivalent. (A) Jak2 with SH2-Bβ bound has catalytic rate constants that are 10 times greater than the base values (i.e., kphos,slow = 60 min−1, kphos,fast = 600 min−1). Under those conditions, there is no significant effect on Jak2 phosphorylation (compare to Figure 3), because the catalytic activity is already high enough so that two Jak2 molecules remain almost fully phosphorylated on Y1 and Y2 while in the same receptor complex. (B) Jak2 without SH2-Bβ bound has catalytic rate constants that are 10 times lower than the base values (i.e., kphos,slow = 0.6 min−1, kphos,fast = 6 min−1). In this case, Jak2 is not sufficiently phosphorylated on Y1, the SH2-Bβ binding site, and therefore does not enjoy the benefit of SH2-Bβ enhancement of Jak2 catalytic activity. (0.23 MB PDF) [file pcbi.1000364.s002.pdf]

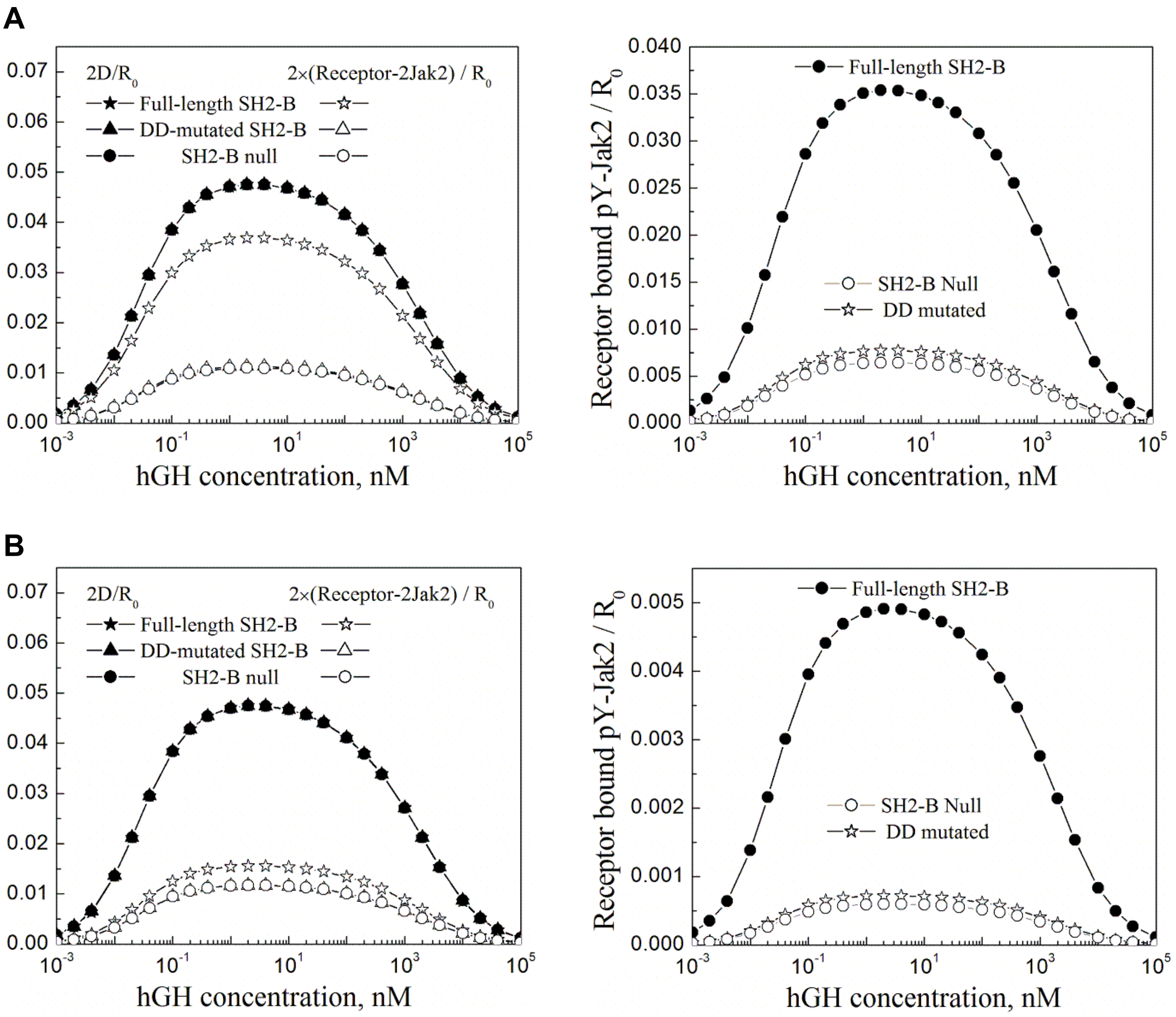

FIGURE S2. Considering the enhancement of Jak2 catalytic activity by SH2-Bβ binding. Calculations were performed using the Simplified Cellular Model as outlined under Fig. 3, except an additional effect was added: Jak2 with SH2-Bβ bound was assumed to have 10-fold higher catalytic activity towards other Jak2 molecules in the same complex. Note here that the absence of SH2-Bβ (SH2-B null) and the inability of SH2-Bβ to dimerize (DD-mutated) are not equivalent. **A.** Jak2 with SH2-Bβ bound has catalytic rate constants that are 10 times greater than the base values (i.e., $k_{phos,slow}$ = 60 min^{-1}, $k_{phos,fast}$ = 600 min^{-1}). Under those conditions, there is no significant effect on Jak2 phosphorylation (compare to Fig. 3), because the catalytic activity is already high enough so that two Jak2 molecules remain almost fully phosphorylated on Y1 and Y2 while in the same receptor complex. **B.** Jak2 without SH2-Bβ bound has catalytic rate constants that are 10 times lower than the base values (i.e., $k_{phos,slow}$ = 0.6 min^{-1}, $k_{phos,fast}$ = 6 min^{-1}). In this case, Jak2 is not sufficiently phosphorylated on Y1, the SH2-Bβ binding site, and therefore does not enjoy the benefit of SH2-Bβ enhancement of Jak2 catalytic activity.
