# Supplementary material for: A Bipolar Clamp Mechanism for Activation of Jak-Family Protein Tyrosine Kinases
Source: PLoS Comput Biol. 2009 Apr 17;5(4):e1000364. doi: 10.1371/journal.pcbi.1000364 (PMC2667146; doi:10.1371/journal.pcbi.1000364)
Supplement: Table S1 — Model parameters. (0.09 MB PDF) [file pcbi.1000364.s003.pdf]

Parameter	Description	Minimum value	Base value (if not varied)	Maximum value
$k_{on,RJ}$	Association rate constant, receptor-Jak2	—	0.06 $nM^{-1}min^{-1}$	—
$K_{D,RJ} = k_{off,RJ}/k_{on,RJ}$	Dissociation constant, receptor-Jak2	10 nM [a]	100 nM	100 nM
$k_{on,JS}$	Association rate constant, Jak2-SH2-Bβ	—	0.06 $nM^{-1}min^{-1}$	—
$K_{D,JS} = k_{off,JS}/k_{on,JS}$	Dissociation constant, Jak2-SH2-Bβ	1 nM [b]	100 nM	100 nM
$k_{on,SS}$	Association rate constant, SH2-Bβ dimerization	—	0.06 $nM^{-1}min^{-1}$	—
$K_{D,SS} = k_{off,SS}/k_{on,SS}$	Dissociation constant, SH2-Bβ dimerization	0.1 nM [b]	100 nM	10 μM
$k_{on,SP}$	Association rate constant, phosphoinositide-SH2-Bβ	—	0.06 $nM^{-1}min^{-1}$	—
$K_{D,SP} = k_{off,SP}/k_{on,SP}$	Dissociation constant, phosphoinositide-SH2-Bβ	—	100 nM	—
J_{Tot}	Total Jak2 concentration	14 pM [b]	100 nM	100 nM
S_{Tot}	Total SH2-Bβ concentration	0.01 nM [b]	100 nM	100 μM [b]
P_{Tot}	Total phosphoinositide concentration	0	1 μM	1 μM
$k_{phos,slow}$	Jak2 transphosphorylation rate constant (Y2 of the kinase not phosphorylated)	—	6 min^{-1}	—
$k_{phos,fast}$	Jak2 transphosphorylation rate constant (Y2 of the kinase phosphorylated)	—	60 min^{-1}	—
k_{dephos}	Jak2 dephosphorylation rate constant	—	6 min^{-1}	—
χ_m	Enhancement factor for interactions at the membrane	—	167	—
χ_r	Effective intra-complex concentration	—	100 μM	—

Table S1. Model parameters. Growth hormone-receptor binding, receptor dimerization, and receptor trafficking parameters, not listed here, are from *Biotechnology Progress*, 20: 1337-1344 (2004). [a] Results not shown. [b] Extreme values considered for the In Vitro Model only.
